# Edaravone (MCI-186) is effective as a free radical scavenger following arteriovenous sheathotomy for treatment of macular oedema associated with branch retinal vein occlusion

**DOI:** 10.1136/bjo.2008.154930

**Published:** 2009-08-09

**Authors:** T Maeno, R Tano, H Takenaka, T Mano

**Affiliations:** 1 Tane Memorial Eye Hospital, Osaka, Japan; 2 Department of Ophthalmology, Osaka Medical College, Takatsuki, Japan

## Abstract

**Aims:**

To determine whether edaravone (MCI-186), a free radical scavenger, can reduce macular oedema and improve the visual acuity after arteriovenous sheathotomy in eyes with a branch retinal vein occlusion (BRVO).

**Methods:**

Forty-seven eyes of 47 consecutive patients with a BRVO who were treated with arteriovenous sheathotomy were studied. The patients were assigned prospectively to either Group R who received 30 mg of edaravone (Radicut) systemically during the vitrectomy or Group N who did not receive any drugs. The postoperative visual acuity was measured before and 12 months after the operation.

**Results:**

At 12 months postoperatively, the best-corrected visual acuity (BCVA) in logarithm of the minimum angle of resolution (logMAR) units improved significantly from 0.22 to 0.56 logMAR units in Group R and from 0.20 to 0.27 units in Group N (p = 0.016). Twenty-three of 27 cases (85%) in Group R and four of 15 cases (27%) in Group N showed an improvement in BCVA of >0.2 logMAR units (p = 0.0025).

**Conclusion:**

The better visual acuity in patients given edaravone than those without endaravone during the arteriovenous sheathotomy suggests that edaravone improved the physiology of the retinal cells after the arteriovenous sheathotomy.

Earlier studies have shown that vitrectomy is effective in reducing the macular oedema associated with a branch retinal vein occlusion (BRVO).^1^ ^2^ The surgical procedures used to treat a BRVO during vitrectomy have been broadened to include creating a posterior vitreous detachment,^3^ removal of the inner-limiting-membrane^4^ and administration of intravitreal triamcinolone acetonide (TA).^5^ It has been proposed that the reduction of macular oedema by vitrectomy was due to the release of vitreal traction on the retina and an enhancement of the cytokine metabolism.^6^ TA and anti-vascular endothelial growth factor (VEGF) have been given to reduce the permeability of the retinal veins caused by the BRVO.

A more recent adjunctive procedure used during the vitrectomy is arteriovenous sheathotomy. The rationale for this procedure is that it reduces or eliminates the pressure on the retinal vein at the arteriovenous crossing caused by the BRVO.^7^ ^8^ In addition, it was expected that a combination of arteriovenous sheathotomy and vitrectomy would result in a greater reduction in BRVO-associated macular oedema.^9^ However, the results showed that the postoperative visual acuity was not significantly improved.^10^ Even in studies in which the reperfusion of the occluded retinal vein was accomplished, a reduction of macular oedema and an improvement of the visual acuity were not always obtained.^11^

Free radicals are often produced in patients with a cerebral infarction, and the free radicals can then damage endothelial cells and neurocytes during the reperfusion.^12^ ^13^ A recent study showed that hydroxyl radicals were generated in the retina during an ischaemic episode and remained elevated during the reperfusion period.^14^ ^15^ These hydroxyl radicals can be inactivated by reactive oxygen species scavengers, such as superoxide dismutase and catalase.^16^

Edaravone (3-methyl-1-phenyl-2-pyrazolin-5-one), a free radical scavenger, was developed in Japan as a neuroprotective agent against the free radicals generated by ischaemia.^17^ Edavarone is an electron donor and when it interacts with free radicals, the reaction yields a peroxyl anion and an edaravone radical, which is then transformed into 4,5-dione. The hydrolysis of 4,5-dione gives 2-oxo-3-phenylhydrazono-butanoic acid.^18^ Edaravone blocks both the water-soluble and lipid-soluble peroxyl radical-induced peroxidation systems.

Arteriovenous sheathotomy for the treatment of BRVO generates free radicals during the reperfusion that may damage the retinal cells and affect the postoperative visual acuity. Thus, the purpose of this study was to determine whether a systemic administration of edaravone during vitrectomy would enhance the recovery of vision after arteriovenous sheathotomy for the treatment of macular oedema associated with BRVO.

## Materials and methods

Forty-seven eyes of 47 consecutive patients who had undergone vitrectomy for BRVO combined with arteriovenous sheathotomy between February 2003 and December 2005 in Tane Memorial Hospital, Osaka, Japan, were studied. All patients had macular oedema with or without a haemorrhage in the macular region accompanying the BRVO, and had a preoperative best-corrected visual acuity (BCVA) ⩽20/40. Preoperative fluorescence angiography (FA) showed fluorescein dye leakage from the vein at the arteriovenous crossing in all patients. There were 24 men and 23 women, ranging in age from 41 to 78 years. The best estimated interval between the onset of the BRVO onset and the initial examination was 4 to 50 months.

Before the surgery, the procedures to be used and the possible consequences of the surgery were explained to each patient. The patients were assigned to two groups prospectively: Group R consisted of 30 consecutive patients who were treated between February 2003 and December 2004 and received 30 mg edaravone; Group N consisted of 17 consecutive patients who were treated between January 2005 to December 2005 and did not receive any drugs. In Group R, 30 mg of edaravone was dissolved in 100 ml of sterile physiological saline and administered intravenously over a period of 2 h at the beginning of surgery.

**Figure 1:**
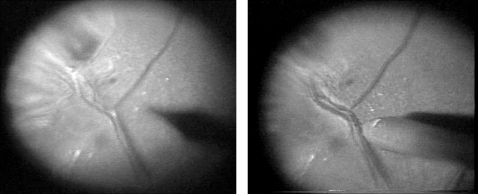
Intraoperative photographs of the fundus in an eye with a branch retinal vein occlusion before (left) and after (right) arteriovenous sheathotomy. A restoration of blood flow after the arteriovenous sheathotomy can be seen.

A standard 20 gauge, three-port vitrectomy was performed with an intentional posterior vitreous separation using a vitreous cutter in eyes without a posterior vitreous detachment. Arteriovenous sheathotomy was performed at the arteriovenous crossing with a BRVO knife (DORC Co., Ltd., Zuidland, Holland; fig 1). All eyes were treated by the same surgeon (T M). None of the patients was given TA before, during or after the surgery. Photocoagulation was not used to treat any of the patients. All of the patients were phakic and had only a mild cataract that did not affect the preoperative visual acuity. The inner limiting membrane was not removed, and none of the patients underwent a cataract operation postoperatively. None of the patients required a gas tamponade. At the end of the surgery, all eyes were treated with a topical antibiotic ointment and 0.1% fluorometholone for 4 weeks. On days 1 and 2 after the surgery, edaravone was given intravenously over a 2 h period twice a day as done preoperatively to the patients in Group R.

There were no significant differences in the estimated duration of the BRVO, average age or sex between the two groups. The mean observation period was 11.3 (SD 1.7) months and 42 of the 47 patients were followed for 12 months after the operation. In all patients, the visual acuity and macular oedema as evaluated by leakage of fluorescein during fluorescein angiography were measured before and after the operation, and the preoperative and postoperative results were compared. Before and 12 months after the operation, the assessment of the presence of fluorescein leakage and the degree of macular oedema was made by trained ophthalmologists who were masked to which group the eye belonged. Before and 3 and 12 months after the operation, the vision test was performed by examiners (who were masked to which group the eye belonged to) with a Japanese Snellen chart, and the values were converted to the logarithm of the minimum angle of resolution (logMAR) units.

The visual acuity in logMAR units was used for the statistical analyses. The differences in the mean visual acuity between the two groups were analysed using the Student’s t test. An improvement in the visual acuity was defined as an increase of ⩾0.2 logMAR units, while a decrease was defined as a reduction of ⩾0.2 logMAR units. The difference in the visual acuity improvement ratio between the two groups was analysed using the Mann–Whitney U test. The correlation between the interval between the onset of symptoms and the surgery and improvement of visual acuity was determined by the Kruskal–Wallis test. Differences in the reduction in macular oedema between the two groups were analysed using Fisher’s exact probability test.

## Results

The intravenous edaravone did not cause any significant side effects in any of the patients. During the follow-up period, none of the patients developed a secondary cataract that affected the visual acuity in both groups. The correlation between the interval between the onset of symptoms and the surgery and the improvement of visual acuity was not significant (p = 0.30). The artery and vein at the arteriovenous crossing was separated by the sheathotomy in all subjects. An increase in the blood flow through the occluded vein postoperatively was confirmed in all subjects by fluorescein angiography at the last examination.

**Figure 2:**
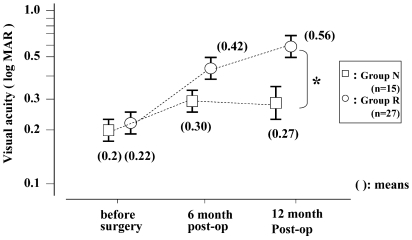
Changes in the best-corrected visual acuity in logarithm of the minimum angle of resolution (logMAR) units before the operation, and at 3 months and 12 months postoperatively. Vertical bars represent SEM. *p<0.05, Student’s t test.

In Group R, the mean BCVA was 0.22 (SD 0.05) logMAR units before the operation, and it improved to 0.42 (SD 0.05) logMAR units at 3 months and 0.56 (SD 0.07) logMAR units at 12 months after the operation. In Group N, the mean BCVA was 0.20 (SD 0.07) logMAR units before the operation, 0.30 (SD 0.07) logMAR units at 3 months, and 0.27 (SD 0.11) logMAR units at 12 months postoperatively. The difference in the preoperative mean visual acuity between the two groups was not significant, but at 12 months after the surgery the mean visual acuity was significantly better in Group R than in Group N (p = 0.086 at 3 months; p = 0.016 at 12 months; fig 2).

**Figure 3:**
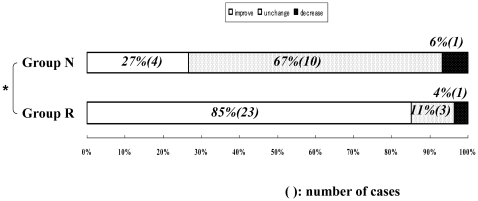
The ratio of the visual acuity improvement between Group N and Group R. The improvement in visual acuity was defined as an increase of ⩾0.2 logarithm of the minimum angle of resolution (logMAR) unit, while a decrease was defined as a reduction of >0.2 logMAR units. *p<0.01, Mann–Whitney U test.

At 12 months postoperatively, 23 of 27 eyes (85%) in Group R had an improvement in the visual acuity ⩾0.2 logMAR units, three eyes (11%) were unchanged, and one eye (4%) had a decrease of >0.2 logMAR units. In the 15 eyes in Group N, four eyes (27%) showed an improvement of ⩾0.2 logMAR units, ten eyes (67%) were unchanged, and one eye (6%) had a decrease of >0.2 logMAR units in the visual acuity. The percentage of eyes with an improvement of visual acuity rate in Group R was significantly higher in Group R than in Group N (p = 0.0025; fig 3).

**Figure 4:**
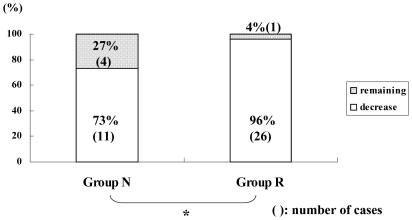
Ratio of reduced to unchanged macular oedema at 12 month postoperatively in Group N and Group R. *p<0.05, Fisher’s exact probability test.

Macular oedema was reduced in patients whose fluorescence angiogram showed a reduction in postoperative leakage. Using this definition for the 42 eyes that were followed for at least 12 months after the operation, there was a decrease in the macular oedema in 26 (96%) of 27 eyes in Group R, and in 11 (73%) of 15 eyes in Group N. This difference was significant (p = 0.047; fig 4).

## Discussion

There are conflicting results on the effectiveness of vitrectomy against the macular oedema in eyes with a BRVO. Thus, an earlier study reported that vitrectomy alone led to a reduction in macular oedema and an improvement in visual acuity,^2^ and later clinical reports have also reported that arteriovenous sheathotomy was effective as an adjunct to vitrectomy in reducing macular oedema.^7^ ^8^ However, there are also some reports that the decrease in macular oedema was not significant, or a significant improvement of visual acuity was not attained although a decrease of the macular oedema was obtained.^10^

In eyes without a retrograde blood flow in the occluded vein or without a collateral pathway preoperatively, arteriovenous sheathotomy led to a restoration of blood flow downstream of the arteriovenous crossing, ie a reperfusion. Even with an improvement of venous blood flow, two factors need to be considered to explain the macular oedema was not reduced and the visual acuity not improved. First, the photoreceptor and neural cells in the inner retina could have been irreversibly damaged during the period of occlusion. Second, reperfusion can generate free radicals that can damage neuronal and vascular endothelial cells. These factors need to be considered when assessing the efficacy of arteriovenous sheathotomy.

In our cases, the preoperative visual acuity, estimated duration of the BRVO and degree of macular oedema between the two groups were not significantly different. These preoperative findings indicate that the physiological condition of the photoreceptors and other retinal neurons was probably similar in the two groups. However, our results showed that the improvements in both visual acuity and macular oedema were significantly better in patients in Group R than that in Group N. This suggests that the decrease in these functions was due to the effects of free radicals produced during the reperfusion.

It has been reported that the concentration of intracellular calcium ions rises in the ischaemic retina, leading to successive catalysis of calpaine, phospholipase A2 and nitric oxide synthase,^19^ ^20^ ^21^ ^22^ and finally the production of superoxide anion, hydrogen peroxide and hydroxyl radical during the reperfusion.^15^ In our cases, the arteriovenous sheathotomy can be considered to be the procedure that led to the reperfusion and the generation of the free radicals.

Edaravone was developed as an effective eliminator of the free radicals produced at the acute stage of cerebral infarction that would protect the ischaemic brain from oxidative damage.^17^ Thus, the mechanism of edaravone treatment is different from the conventional anticoagulant, platelet-aggregation inhibitor and anti-VEGF treatments used in cases of vascular blockage. Edaravone has a molecular mass of 174.20 Da and eliminates hydroxyl radicals with strong oxidative power, as well as water-soluble and liposoluble peroxyl radicals. This property of edaravone arises because it is a free radical scavenger of the electron-donor type. In contrast, the antioxidants vitamins E and C, which act by donating a hydrogen atom, eliminate only peroxyl radicals. Consequently, edaravone is considered to be more effective as an eliminator of free radicals.^23^

The patients in Group R who received 30 mg edaravone at the start of the vitrectomy had significantly better visual acuity at the final examination. This strongly suggests that the improvement was caused by the reduction of free radical-induced damage to neuronal cells and vascular endothelial cells.^24^ ^25^ The reduction of macular oedema was caused by the prevention of the damage to the vascular endothelial cells at the early stage after the reperfusion.

It generally requires several months for the visual acuity to improve after vitrectomy for vitreoretinal diseases. In our cases, a statistically significant improvement in the mean visual acuity was detected at 12 months after the surgery in Group R. However, the difference in the mean visual acuity at 3 months between Group R and Group N was not significant, which also suggests that a longer time is required for visual acuity improvement.

There are several obvious limitations to this study. First, the number of patients was small. Second, 30 mg of edaravone was given for only 2 days: extending the duration of edavarone treatment might have led to a greater improvement of the visual acuity. In addition, combined use of edaravone with conventional anticoagulants, platelet-aggregation inhibitors or anti-VEGF treatments might also have additive and synergistic effects.

In conclusion, vitrectomy combined with arteriovenous sheathotomy in eyes with macular oedema due to BRVO appeared to lead to better results then when edaravone was given intravenously at the time of the vitrectomy. Further studies that address the limitations discussed are necessary to confirm the findings in this study. Edaravone should be given as early as possible to prevent irreversible change to photoreceptors and retinal neurons.
